# SARS-CoV-2 infection in pregnant women assisted in a high-risk maternity hospital in Brazil: Clinical aspects and obstetric outcomes

**DOI:** 10.1371/journal.pone.0264901

**Published:** 2022-03-11

**Authors:** Carolina Loyola Prest Ferrugini, Neide Aparecida Tosato Boldrini, Franco Luis Salume Costa, Michelle Anne de Oliveira Batista Salgueiro, Pamella Dunga de Paula Coelho, Angelica Espinosa Miranda

**Affiliations:** 1 Federal University of Espírito Santo, Post-Graduation Program in Infectious Diseases, Vitória, ES, Brazil; 2 Department of Gynecology and Obstetrics, Federal University of Espírito Santo, Vitória, ES, Brazil; 3 Cassiano Antonio Moraes University Hospital, Maternal-Child Unit, Vitória, ES, Brazil; 4 Federal University of Espírito Santo, Medical Residency Program in Gynecology and Obstetrics, Vitória, ES, Brazil; University of Cape Town, SOUTH AFRICA

## Abstract

**Background:**

The spread of severe acute respiratory syndrome coronavirus 2 (SARS-CoV-2) infection, the possible development of serious illness, and the possibility of severe obstetric outcomes highlight the importance of addressing SARS-CoV-2 infection in obstetric management.

**Methods and findings:**

A cross-sectional study of pregnant women assisted in a high-risk maternity hospital in Brazil in 2020. All patients admitted for delivery or miscarriage care were tested for SARS-CoV-2 using polymerase chain reaction (PCR) and for immunoglobulin (I)gM, and/or IgG by immunochromatography. Clinical aspects and obstetric outcomes were analyzed. A total of 265 pregnant women were included in the study. There were 38 (14.4%) PCR positive cases during pregnancy, 12 (31.6%) on admission screening, and 71(27.2%) patients were IgM- and/or IgG-positive. Among the participants, 86 (32.4%) had at least one positive test during pregnancy. SARS-CoV-2 positive patients had greater contact with known positive patients (p = 0.005). The most frequently reported symptoms were runny nose, cough, loss of smell and taste, headache, and fever. There was also a 35% rate of asymptomatic infections and a 4.6% rate of severe or critical infections. Patients exposed or infected with SARS-CoV-2 had a higher incidence of preterm delivery, cesarean section, need for resuscitation in the delivery room, Apgar score <7 at 5 min, admission to the neonatal intensive care unit, and jaundice. Newborns with at least one positive test had a significantly greater need for phototherapy after delivery (p = 0.05). The results showed a high rate of positive tests among newborns (37.5%), which seems to be compatible with both neonatal and perinatal infection.

**Conclusions:**

It is important to further investigate SARS-CoV-2 infection during pregnancy, including the clinical course and the possibility of adverse outcomes with impact on maternal and fetal health, regardless of the development of symptoms.

## Introduction

In January 2020, the World Health Organization declared a public health emergency due to coronavirus disease 2019 (COVID-19). The disease, caused by severe acute respiratory syndrome coronavirus 2 (SARS-CoV-2), has challenged all health systems and has caused an unprecedented global public health crisis [1]. SARS-CoV-2, unlike other severe acute respiratory syndrome (SARS) viruses responsible for previous epidemics, has been transmitted by oligo- or asymptomatic patients, challenging previously implemented quarantine strategies [2]. Such reports of asymptomatic transmission led medical services to implement universal screening policies for certain groups of patients, including pregnant women admitted for delivery [3]. The identification of infected patients helps health services implement appropriate isolation practices and evaluate the impact of infection on obstetric and perinatal outcomes.

Data on SARS-CoV-2 infection during pregnancy have been extensively studied, but scientific consensus has yet to be achieved [4]. Different findings regarding obstetric and neonatal consequences of this infection during various phases of pregnancy highlight the need to better understand this specific population [5–7]. An observational cohort study including 683,905 women from 2022 showed that SARS-CoV-2 infection during pregnancy was associated with preterm birth, preeclampsia, and several other clinical complications, but was not associated with a higher incidence of stillbirths [4]. Another 2021 retrospective cohort study that included 850,364 women showed an increased rate of premature birth but no significant increase in cesarean delivery [8]. On the other hand, earlier studies failed to associate SARS-CoV-2 infection to adverse pregnancy outcomes, including premature birth, cesarean delivery and fetal or neonatal death [9, 10]. Furthermore, data from SARS-CoV-2 infection in pregnant women in Brazil are limited to a case series [11].

The objectives of this study were to determine the rate of positive RT-PCR tests and/or positive antibody tests (IgG/IgM) for SARS-CoV-2 during pregnancy in patients admitted to the Cassiano Antônio Moraes University Hospital (HUCAM) maternity hospital for childbirth or abortion care, evaluate the most commonly reported symptoms in patients with positive test results for SARS-CoV-2, evaluate the rate of the main obstetric and neonatal outcomes in positive antibody patients and/or positive RT-PCR patients when compared with non-infected pregnancies (PCR and IgG/IgM negative), and to assess the positivity rate in newborns of mothers with recent infection (PCR positive and/or IgM positive and IgG negative on admission for delivery).

Access to clinical and epidemiological data of obstetric patients seen at the maternity ward at HUCAM, from Vitória, Espírito Santo, Brazil, during the pandemic helps to clarify conflicting data about SARS-CoV-2 infection during pregnancy.

## Methods

This was a cross-sectional study conducted with pregnant women who were assisted at the HUCAM high-risk maternity unit from July to October 2020.

HUCAM has a reference maternity hospital for pregnant women with high obstetric risk in Vitória, capital of Espírito Santo, southeast, Brazil. High obstetric risk includes, but is not exclusive to, patients infected with SARS-CoV-2. All patients admitted to the unit for delivery or abortion care were tested for SARS-CoV-2 using PCR, IgG, and/or IgM regardless of the presence of symptoms. Clinical aspects and obstetric outcomes were analyzed.

Upon admission for childbirth or abortion care, patients were asked about their history of SARS-CoV-2 infection in their current pregnancy. Those with a positive PCR test for SARS-CoV-2 during pregnancy were offered serology by immunochromatography. Patients without a history of documented SARS-CoV-2 infection during pregnancy were offered a nasal and oropharyngeal RT-PCR swab and immunochromatographic test for IgG and IgM using blood samples obtained by fingertip puncture. We used a commercial immunochromatographic assay (Eco teste® from Eco Diagnóstica®) validated by Brazil’s national health surveillance agency (ANVISA) with reported sensitivity of 95% and 90% for IgG and IgM, respectively and specificity of 99% and 94%, respectively [12]. Newborns of PCR-positive patients or IgM-positive IgG-negative patients on admission were also tested by PCR.

The patients answered a questionnaire which contained information on age, education, profession, marital status, self-reported skin color, weight, height, smoking, alcohol and illicit drug use, chronic diseases, number of people in the household, contact with known positive patients, interstate or international travel, social isolation, presence of symptoms potentially associated with COVID-19 (fever, cough, shortness of breath, sore throat, headache, asthenia, myalgia, diarrhea, sneezing, runny nose, and/or smell and taste disorders).

Patients with a positive PCR test prior to hospital admission were asked about the presence of symptoms at the time of diagnosis. Patients with a positive PCR test and/or negative IgG and IgM on admission were asked about the presence of current symptoms. Finally, the remaining patients were asked about the presence of any symptoms since March, when the pandemic started.

Social isolation was defined as complying with government guidelines to leave the house to perform only strictly necessary activities. Patients who worked in essential activities and needed to maintain their routine work activities were considered not to have to complied with social isolation.

Body weight was classified according to the body mass index per week of pregnancy on the date of admission, as recommended by the Ministry of Health Brazil, 2011 [13].

Pregnancy and obstetric outcome data were analyzed, including gestational age, parity, presence of pre-eclampsia or gestational diabetes, normal term delivery or cesarean section, premature delivery, miscarriage or fetal loss, ectopic pregnancy, molar pregnancy, appropriate newborn weight for gestational age, delivery room resuscitation, neonatal death, Apgar index, neonatal intensive care unit (NICU) admission, neonatal jaundice, and phototherapy.

These data were included in the SPSS software for Windows (version 21.0, SPSS Inc., Chicago, IL, USA) for statistical analysis. Initially, descriptive analysis included frequency distribution for qualitative variables and median and interquartile range for quantitative variables. Categorical variables were analyzed using the relative frequency. The chi-square or Fisher’s exact test was used to measure the association between independent categorical variables and cases diagnosed with SARS-CoV-2. The dependent variable was the diagnosis of SARS-CoV-2 during pregnancy. The crude and adjusted logistic regression model odds ratios (ORs) were calculated for the variables that showed a statistical significance of 20% in the chi-square test. The adjusted ORs of the variables remaining in the final model are presented. The final significance level was 5%.

This study was approved by the HUCAM Research Ethics Committee (number 32490720.0.0000.5071/2020). The information collected was used only in the present study. The confidentiality of information was protected so that the identities of the pregnant women were safeguarded. Written informed consent was obtained from all patients and the parents or guardians of the minors included in the study.

## Results

A total of 265 pregnant women were admitted to HUCAM from July to October 2020 and were included in the study. Of them, 263 underwent nasal and oropharyngeal PCR during pregnancy or delivery and 261 underwent IgG and IgM detection by immunochromatography. Two patients underwent antibody collection only, and four patients underwent PCR only on admission. Eight of them authorized testing their newborns.

Of all the included patients, 86 (32.4%) had at least one positive test during pregnancy, including 38 (14.4%) PCR-positive cases during pregnancy (31.6% on admission screening), and 71 (27.2%) IgG and/or IgM positive.

The demographic, clinical and behavioral data of pregnant women, grouped according to the type of positive test, are presented in Table 1. Most of the patients were aged 20 to 34 years old, had completed their high school education (p = 0.04 for presence of antibodies). The association between obesity and SARS-CoV-2 infection was statistically significant for at least one positive test (p = 0.038) and the presence of antibodies (p = 0.040). Also, SARS-CoV-2-positive patients had greater contact with known positive patients (p = 0.001 for at least one positive test).

**Table 1:** Sociodemographic, clinical and behavioral data of pregnant women by test type and result (N = 265).

N (%)	At least one + test (n = 86)	p	PCR + (n = 38)	p	IgM/IgG + (n = 71)	p	PCR—and IgG/IgM—(n = 179)
**Age (y)**							
**≤19 y old**	13 (15.1)	0.49	5 (13.2)	0.77	11 (15.5)	0.47	18 (10.1)
**20–34 y old**	57 (66.3)		27 (71.1)		48 (67.6)		126 (70.4)
**≥35 y old**	16 (18.6)		6 (15.8)		12 (16.9)		35 (19.6)
**Education**							
**<5 y**	10 (11.6)	0.54	7 (18.4)	0.77	5 (7)	0.04	27 (15.1)
**Elementary school**	28 (32.6)		7 (18.4)		28 (39.4)		44 (24.6)
**High school**	41 (47.7)		20 (52.6)		35 (49.3)		83 (46.4)
**Higher education**	7 (8.1)		4 (10.5)		3 (4.2)		24 (13.4)
**Master’s degree**	0 (0)		0 (0)		0 (0)		1 (0.6)
**Employed**	36 (41.9)	0.12	18 (47.4)	0.59	29 (40.8)	0,09	94 (52.5)
**Living with a partner**	64 (74.4)	0.20	31 (81.6)	0.08	51 (71.8)	0.39	119 (66.5)
**Ethnicity**							
**Black**	75 (87.2)	0.22	35 (92.1)	0.22	62 (87.3)	0.36	148 (82.7)
**Other**	11 (12.8)		3 (7.9)		9 (12.7)		31 (17.3)
**BMI**							
**Low or normal weight**	24 (27.9)	0.04	11 (28.9)	0.17	19 (26.8)	0.04	73 (41)
**Overweight or**	62 (72.1)		27 (71.1)		52 (73.2)		105 (59)
**Obese**							
**Smoking**	3 (3.5)	0,87	0 (0)	0.21	3 (4.2)	0.9	7 (3.9)
**Alcohol or drug user**	3 (3.5)	0,36	0 (0)	0.12	3 (4.2)	0.55	11 (6.1)
**Chronic illness** *	29 (33.7)	0,5	14 (20.9)	0.38	26 (36.6)	0.29	53 (29.6)
**Number of people per household**							
**≤2**	26 (30.2)	0,05	12 (31.6)	0.74	22 (31)	0.65	52 (29.1)
**3–5**	50 (58.1)		23 (60.5)		38 (53.5)		105 (58.7)
**≥6**	10 (11.6)		3 (7.9)		11 (15.5)		22 (12.3)
**Contact with + patient**	27 (31.4)	0.001	20 (52.6)	0.00	20 (28.2)	0.009	25 (14)
**Interstate travel**	3 (3.5)	0.36	2 (5.3)	0.84	2 (2.8)	0.28	11 (6.1)
**Social isolation**	62 (72.1)	0.06	23 (60.5)	0.98	52 (73.2)	0.05	108 (60.3)
**Chronic illness** *	29 (33.7)	0,5	14 (20.9)	0.38	26 (36.6)	0.29	53 (29.6)

PCR: Viral detection by Reverse Transcription Polymerase Chain Reaction; BMI: Body Mass Index

*Previous chronic diseases: diabetes, hypertension, cardiovascular, kidney, or lung disease.

Table 2 presents the main data on gestational age, number of pregnancies, and the presence of pre-eclampsia and gestational diabetes.

**Table 2:** Obstetric data by test type and result (N = 265).

N (%)	At least one + test (n = 86)	p	PCR +(n = 38)	p	IgM/IgG +(n = 71)	p	PCR—and IgG/IgM—(n = 179)
**Gestational age**							
**≤13 w 6 d**	3 (3.5)	0.20	1 (2.6)	0.68	3 (4.2)	0.27	14 (7.8)
**14 w– 27 w 6 d**	2 (2.3)		2 (5.3)		1 (1.4)		8 (4.5)
**28 w– 36 w 6 d**	20 (23.3)		7 (18.4)		16 (22.5)		27 (15.1)
**≥37 w**	61 (70.9)		28 (73.7)		51 (71.8)		130 (72.6)
**Primiparous**	23 (26.7)	0.77	7 (18.4)	0.20	20 (28.2)	0.94	51 (28.5)
**Pre-eclampsia**	19 (22.1)	0.42	7 (18.4)	0.94	18 (25.4)	0.19	32 (17.9)
**Gestational diabetes**	27 (31.4)	0.69	12 (31.6)	0.76	20 (28.2)	0.87	52 (29.1)

PCR: Viral detection by Reverse Transcription Polymerase Chain Reaction.

Only the variable “contact with known positive patients” remained associated with SARS-CoV-2 infection in the final logistic regression model (Table 3).

**Table 3:** Final model of logistic regression of covariates associated with SARS-CoV-2 diagnostic test.

Any positive Test				
Parameter	OR	CI 95%	Adjusted OR	Adjusted CI 95%
Employed	0,651	(0,387–1,094)	0,668	(0,366–1,214)
BMI	0,557	(0,319–0,973)	0,661	(0,356–1,227)
Contact with + patient	2,819	(1,515–5,247)	2,629	(1,342–5,154)
Social isolation	1,698	(0,972–2,968)	1,599	(0,824–3,103)
Preterm birth	1,555	(0,821–2,945)	1,498	(0,646–3,471)
Adequacy weight for the GA	*		1,045	(0,586–1,863)
Neonatal ICU admission	1,6	(0,874–2,927)	1,029	(0,474–2,235)
Phototherapy	1,922	(0,994–3,716)	1,556	(0,708–3,420)
**PCR positive tests**				
BMI	0,586	(0,273–1,256)	0,863	(0,362–2,056)
Contact with + patient	5,544	(2,582–11,905)	4,239	(1,845–9,742)
Preterm birth	1,291	(0,534–3,120)	1,215	(0,388–3,813)
Adequacy weight for the GA	*		1,258	(0,555–2,850)
Phototherapy	1,595	(0,650–3,915)	1,307	(0,438–3,899)
Living with a partner	2,233	(0,929–5,367)	1,722	(0,666–4,454)
Alcohol or drug user	0,816	(0,764–0,870)	0,000	(0,000-)
Apgar score < 7 at 5’	0,355	(0,081–1,559)	0,178	(0,023–1,378)
**Antibody positive test**				
BMI	0,533	(0,291–0,976)	0,667	(0,344–1,295)
Contact with + patient	2,4	(1,231–4,681)	2,360	(1,125–4,951)
Phototherapy	1,739	(0,855–3,537)	1,488	(0,643–3,447)
Apgar score < 7 at 5’	2,143	(0,246–18,699)	1,595	(0,083–30,741)
Employed	0,617	(0,353–1,077)	0,668	(0,344–1,300)
Education	*		0,889	(0,601–1,315)
Social Isolation	1,816	(0,992–3,326)	1,732	(0,825–3,635)
Preeclampsia	1,550	(0,803–2,990)	1,329	(0,647–2,731)
NB resuscitation in the delivery room	1,599	(0,778–3,285)	1,422	(0,606–3,335)
Neonatal death	0,698	(0,640–0,761)	0,000	(,000-)
Neonatal ICU admission	1,539	(0,806–2,939)	1,201	(0,544–2,650)

Table 4 presents the main symptoms reported by SARS-CoV-2 positive patients per test. Thirty percent of the patients with at least one positive test for SARS-CoV-2 reported no flu symptoms during any period of their pregnancy. Of the patients reporting symptoms, most had mild clinical symptoms and only six required hospital admission due to respiratory symptoms, two with moderate symptoms and four with severe symptoms requiring mechanical ventilation and ICU admission. None of the patients progressed unfavorably. Only one patient required puerperal hysterectomy, and all were discharged without other sequelae. The most frequently reported symptoms, in order of the most-to-least reported, were runny nose, cough, decreased sense of smell and taste, headache and fever. As for the number of symptoms, 7% reported only one, 17% reported two, and 40% reported three or more symptoms. Patients with a positive test were duly evaluated and investigated by the researcher to ensure that all of them had symptoms compatible with SAS-CoV-2 infection during pregnancy, except for one patient who was in early pregnancy and was not sure of the date of the symptoms and their correspondence with the current pregnancy.

**Table 4:** Symptoms suggestive of SARS-CoV-2 infection by test type and result among SARS-CoV-2-positive pregnant women.

	At least one + test n = 86 (%)	PCR + n = 38 (%)	IgM/IgG + n = 71 (%)
**Fever**	25 (29.1)	15 (39.5)	19 (26.8)
**Cough**	28 (32.6)	19 (50)	20 (28.2)
**Shortness of breath**	16 (18.6)	13 (34.2)	10 (14.1)
**Sore throat**	10 (11.6)	8 (21.1)	9 (12.7)
**Headache**	26 (30.2)	18 (47.4)	11 (26.8)
**Asthenia/myalgia**	25 (29.1)	19 (50)	17 (23.9)
**Diarrhea**	8 (9.3)	7 (18.4)	5 (7)
**Sneezing**	12 (14)	7 (18.4)	10 (14.1)
**Runny nose**	30 (34.9)	20 (52.6)	23 (32.4)
**Loss of sense of smell and taste**	26 (30.2)	17 (44.7)	20 (28.2)
**Asymptomatic**	30 (35)	4 (10.5)	30 (42.3)
**1 symptom**	6 (7)	1 (2.6)	5 (7)
**2 symptoms**	15 (17.4)	7 (18.4)	10 (14.1)
**≥3 symptoms**	35 (40.6)	26 (68.4)	26 (36.6)

PCR: Viral detection by Reverse Transcription Polymerase Chain Reaction.

Table 5 presents the main obstetric outcomes grouped according to the test type and results. Although not statistically significant, patients exposed to or infected by SARS-CoV-2 presented a higher incidence of preterm birth, cesarean section, need for resuscitation in the delivery room, Apgar score <7 at 5 min, NICU admission, and jaundice. Newborns with at least one positive test for SARS-CoV-2 had a greater need for phototherapy after delivery, with statistical significance (p = 0.05, in at least one positive test).

**Table 5:** Obstetric outcomes 19 by test type and result (N = 265).

Outcome	At least one + test n = 86 (%)	p	PCR + n = 38 (%)	p	IgG/IgM + n = 71 (%)	p	PCR—and IgG/IgM—(n = 179)
**Premature birth**	21 (25.6)	0.17	8 (22.2)	0,57	15 (22.4)	0.47	2 (18.1)
**Miscarriage or fetal loss**	6 (7)	0.23	4 (10.5)	0.83	4 (5.6)	0.14	21 (11.7)
**Type of delivery**							
**Cesarean section**	57 (69.5)	0.28	24 (66.7)	0.64	45 (67.2)	0.54	100 (62.5)
**Vaginal**	25 (30.5)		12 (33.3)		22 (32.8)		60 (37.5)
**Adequacy weight for the GA**							
**AGA**	65 (79.3)	0.19	29 (80.6)	0.14	52 (77.6)	0.48	112 (70.4)
**LGA**	11 (13.4)		6 (16.7)		9 (13.4)		23 (14.5)
**SGA**	6 (7.3)		1 (0.5)		6 (9)		24 (15.1)
**NB resuscitation in the delivery room**	17 (20.7)	0.28	6 (16.7)	0.83	15 (22.4)	0.19	24 (15.2)
**Neonatal death**	2 (2.4)	0.98	2 (5.6)	0.34	0 (0)	0.19	4 (2.5)
**Apgar score < 7 at 5’.**	3 (1.9)	0.82	3 (8.3)	0.15	1 (1.5)	0.48	3 (3.7)
**NICU admission**	34 (21.5)	0.13	9 (25)	0.65	20 (29.9)	0.19	25 (30.5)
**Neonatal jaundice**	54 (34.2)	0.58	15 (41.7)	0.39	25 (37.3)	0.68	31 (37.8)
**Phototherapy**	24 (15.2)	0.05	8 (22.2)	0.30	16 (23.9)	0.12	21 (25.6)

PCR: Viral detection by Reverse Transcription Polymerase Chain Reaction; GA: Gestational Age; AGA: Adequate for Gestational Age; LGA: Large for Gestational Age; SGA: Small for Gestational Age; NB: Newborn; ICU: Intensive Care Unit.

Table 6 shows positive results for the newborns tested. Only eight were tested, with three (37.5%) being positive: one 72 h postpartum, born from a PCR-negative, IgM-positive, and IgG-negative mother (admitted to NICU), and two 24 h postpartum, one from a PCR-positive, IgG-, and IgM-negative mother (in rooming-in) and another born from a PCR-positive mother (in NICU).

**Table 6:** RT-PCR tests performed in newborns and the correlation with maternal test results (N = 8).

	GA at birth (w)	Maternal PCR	Maternal IgM	Maternal IgG	NB test (PCR)
**1**	23	Positive	Negative	Negative	Negative w/ 24 and 72 h
**2**	38	Positive	Negative	Negative	Positive w/ 24 h
**3**	35	Negative	Positive	Negative	Negative w/ 24 h
**4**	32	Negative	Positive	Negative	Positive w/ 72 h
**5**	37	Positive	Negative	Negative	Negative w/ 24 h
**6**	28	Negative	Positive	Negative	Negative w/ 24 h
**7**	33	Positive	Not performed	Not performed	Positive w/ 24 h
**8**	30	Positive	Not performed	Not performed	Negative

GA: Gestational Age; NB: Newborns.

## Discussion

Our findings showed a high incidence of SARS-CoV-2 infection in high-risk pregnant women admitted to the HUCAM (32.4% with at least one positive test), including 4.5% of the total number of patients who tested positive by PCR on admission for delivery/miscarriage care. Several other studies have evaluated the incidence of SARS-CoV-2 infection during pregnancy through screening on admission, with conflicting results. Sutton et al. found a positive PCR rate of 15.4% on admission, while Fasset et al found 0.43% [3, 14].

It is worth noting that testing strategies influence the estimated prevalence of SARS-CoV-2 infection. In this study, we performed PCR testing with IgM and IgG antibody detection during pregnancy to diagnose patients with asymptomatic or oligosymptomatic infections who could not be tested by PCR in the appropriate period of symptomatology. It is also important to highlight that this study was conducted at the beginning of the pandemic when no vaccine was available. In this case, considering the beginning of community transmission of the disease in the state of Espírito Santo, the pregnancy dating of the patients included, and the data on antibody detection in patients exposed to the virus, it can be inferred that the patients with SARS-CoV-2 antibodies at the time of the outcome were infected during the gestational period [15–17].

Recent studies have shown that pregnant women are more likely to develop asymptomatic SARS-COV-2 infections than non-pregnant women, but this does not exempt them from experiencing obstetric and neonatal complications due to this infection [5, 6, 14]. The most frequent symptoms reported in this study were coryza, cough, loss of smell and taste, headache, and fever, corroborating the findings of Allotey *et al*., who reported fever and cough [7]. There were also 35% asymptomatic infections, while Villar *et al*. reported 41% [18]. Six women required hospital admission due to respiratory symptoms and four required admission to the ICU and mechanical ventilation, resulting in a rate of 6.9% for moderate to severe infections and 4.6% for severe or critical infections among women with any positive test results.

Although there are data on the importance of maternal morbidity and mortality due to severe acute respiratory infections (SARI), including COVID-19, in Brazil [11], there were no reported deaths as a result of SARS-Cov-2 infection in this study, which may be associated with a selection bias, as HUCAM is not the main referral hospital in the network care for SARS-CoV-2 infection in the State of Espírito Santo, Brazil. The HUCAM obstetric center is a high-risk service for other obstetric and clinical issues, with a mean of 80 to 100 deliveries per month. This selection limits the generalization of our results.

It is important to note that the frequency of unfavorable outcomes was higher in the group of patients exposed to the virus, who showed a higher incidence of cesarean section, premature delivery, admission to NICU, need for resuscitation, neonatal jaundice, and need for phototherapy, which despite not being statistically significant, corroborates the literature, similar to the results of Metz *et al*., Chmielewska *et al*., Litman *et al*., and Chinn *et al*., which showed higher rates of maternal death, clinical complications, fetal loss, ectopic pregnancy, maternal depression and premature birth associated to SARS-CoV-2 infection [4, 5, 8, 19].

Regarding the newborns who tested positive, this study showed a high rate of positive tests (3/8: 37.5%), compared to the literature that reported low perinatal positivity rates [18, 20]. To our knowledge, this finding may be related to both neonatal and perinatal infection, including mother to child transmission. Angelidou *et al*. demonstrated a positivity rate of 2.2% in 225 patients tested during hospitalization after birth, while Villar *et al*. found 13% positivity [18, 20]. A limitation of this study was the small number of newborns tested. In addition, only infants who were born to selected mothers were tested (PCR positive, IgM positive, and IgG negative at delivery). Thus, this may overestimate the frequency of positive infants, since not all infants were tested.

It is important to emphasize that the cases studied included the case of a 23-week twin pregnancy in a woman reporting a history of chills and positive PCR on admission which resulted in extreme premature delivery with two stillbirths, without any other associated maternal or fetal morbidity, corroborating the hypothesis that even mild or asymptomatic cases of infection may result in negative obstetric outcomes.

Finally, our results, as well as the most recent data in the literature, emphasize the lack of elucidation of the clinical course and consequences of COVID-19 in pregnancy, as well as the possibility of adverse outcomes with impact on maternal-fetal health. Therefore, greater investments in the scientific community in the clinical and diagnostic investigation of pregnant and parturient women exposed to COVID-19 infection is necessary.

## Supporting information

S1 File Questionário e dados clínicos das pacientes internadas na maternidade do HUCAM-Covid19. Aplyied questionaire in the original language.
(PDF)

S2 File Questionnaire and clinical data of patients hospitalized at the HUCAM-Covid 19 project. Aplyied translated to english questionaire.
(PDF)

S1 Table Gestational age at PCR-positive test and presence of SARS-CoV-2 antibodies. Specifies the gestational age at which each patient was pcr positive for SARS-CoV-2, the IgG and IgM result at hospital admission for the outcome (delivery or miscarriage), and the gestational age at the outcome.
(PDF)

S1 Dataset Study database. (SAV)
