# Supplementary material for: SARS-CoV-2 infection in pregnant women assisted in a high-risk maternity hospital in Brazil: Clinical aspects and obstetric outcomes
Source: PLoS One. 2022 Mar 11;17(3):e0264901. doi: 10.1371/journal.pone.0264901 (PMC8916667; doi:10.1371/journal.pone.0264901)
Supplement: S1 File — Aplyied questionaire in the original language. (PDF) [file pone.0264901.s001.pdf]

Questionário e dados clínicos das pacientes internadas na maternidade do HUCAM- Covid 19.

Nome:____ Reg:________________________
Telefone:____
01. DN: ___/____/______ 02. Idade:________ 03. Município de residência:_____________________
04. Cor da pele: ( ) preta ( ) outra
05. Vive com parceiro:_______________________ 06. Escolaridade:__________________________
07. Número de cohabitantes:___________ 08. Número de idosos cohabitantes:________
09. Atualmente empregada? ___________________________________
10. Comorbidades prévias: ( ) Não ( ) Hipertensão ( ) Outra doença cardiovascular ( ) Diabetes Mellitus ( ) DPOC/asma ( ) Doença renal crônica ( ) Doença imunossupressora ( ) Obesidade ( ) IST ( ) outro____
Peso: ____________ Altura:____________ ( ) Uso álcool ( ) Uso drogas. Qual?_____________
( ) Tabagismo. Quantos/dia________________ Medicamentos? ( ). Quais?__________________
11. Dados obstétricos:
Gestações: _______ Partos: _______ Abortos:_______ Natimortos: __________ Neomortos:_________
Idade Gestacional: __________________ Grupo Sanguíneo: ____________________
Diabetes Mellitus Gestacional: ( ) sim ( ) não
Síndrome hipertensiva da gravidez: ( ) sim ( ) não
Hiperêmes: ( ) sim ( ) não
Rubéola: IgG ( ) IgM ( )
Toxoplasmose: IgG ( ) IgM ( )
Anti HCV: ( ) positivo ( ) negativo
HBsAg: ( ) positivo ( ) negativo
ANTI-HIV: ( ) positivo ( ) negativo
VDRL: ____________________
Outro:____
13. Apresentou algum dos sintomas listados desde março 20?
( ) Febre ou sensação de febre
( ) Tosse
( ) Dificuldade para respirar
( ) Dor de garganta
( ) Dor de cabeça
( ) Astenia/mialgia
( ) Diarreia
( ) Perda de olfato e paladar
( ) Espirros
( ) Coriza
( ) Nenhum desses sintomas

14.1 Se sim, qual a data do início dos sintomas?_________________________________
14.2 Qual a data do fim dos sintomas?____

14. Nos últimos meses (desde março 20) foi atendido por um profissional da saúde?
( ) Sim ( )Não
15. Nos últimos meses (desde março 20) foi internado devido síndrome gripal?
( ) Sim, em enfermaria ( ) Sim, em UTI ( ) Não
16. Foi submetido ao teste do tipo rt-pcr de rna viral para detecção DO SARS-CoV-2?
( ) Não ( ) Sim, Resultado:_______ Se sim, quando?________________________________
17. Teve contato próximo com caso confirmado de covid-19? ( ) sim ( ) não
18. Teve contato próximo com caso suspeito de covid-19? ( ) sim ( ) não
19. Viagem interestadual ou internacional nos últimos meses? ( ) sim ( ) não
21. Estava trabalhando nos últimos meses? ( ) sim ( ) não ( ) trabalho remoto
22. Respeitou o isolamento social orientado pelo governo? ( ) sim ( ) não
23. Exame clínico na admissão:
PA: ___/____mmhg edema: ( ) sim ( )não AFU: ______cm MS: ( ) não ( ) sim ____/10min
Aminiorrexe: ( ) Sim ( )Não
Temp axilar: _____ Freq respiratória: _____ Freq cardíaca_____ SpO2: ______
24. Exames da internação:
Teste rápido covid-19: IgG _______ IgM________
PCR para COVID-19 (ATÉ 48H): ( ) positivo ( ) negativo (.) não coletado
Teste rápido HIV: ( ) positivo ( ) negativo
Teste rápido sífilis: ( ) positivo ( ) negativo
Teste rápido Hepatite B: ( ) positivo ( ) negativo

Teste rápido Hepatite C: (   ) positivo (   ) negativo

25. Desfecho obstétrico: data do parto:____/____/2020
25.1 (   ) parto termo (   ) parto prematuro (   ) aborto (   ) aborto molar (   ) gravidez ectópica
(   ) outro __________________________
25.2 (   ) parto normal espontâneo (   ) parto normal induzido (   ) parto cesariana urgência (   ) parto cesariana eletiva
25.3 Indicação parto (indução/cesariana): ____
25.4 Complicações no parto:____

26. Dados do RN ao nascimento
(   ) AIG (   ) PIG (   ) GIG
Apgar: _____/______/_______ ____
Peso: ____________________ comprimento: _____________ PT: ________ PC: _______ PA:_______
Clampeamento cordão: (   ) oportuno (   ) imediato
Rotura de membranas: (   ) no ato (   ) antes do expulsivo/ato operatório
Tempo de bolsa rota até o parto: ___________
Admissão na UTIN: (   ) sim (   ) não
Malformação: (   ) sim (   ) não Se sim, qual?____
Trauma do RN parto: (   ) sim (   ) não
Morte neonatal: (   ) natimorto (   ) neomorto (   ) não
Hipoglicemia: (   ) sim (   ) não
GS RN: ______ (   )
Icterícia: (   ) sim (   ) não
Fototerapia: (   ) sim (   ) não
Peso na alta: ______________g Dias de vida:__________________
Condições de alta RN: (   ) LM exclusivo (   ) LM + fórmula (   ) fórmula

DATA:_____/______/2020.
