# Supplementary material for: SARS-CoV-2 infection in pregnant women assisted in a high-risk maternity hospital in Brazil: Clinical aspects and obstetric outcomes
Source: PLoS One. 2022 Mar 11;17(3):e0264901. doi: 10.1371/journal.pone.0264901 (PMC8916667; doi:10.1371/journal.pone.0264901)
Supplement: S2 File — Aplyied translated to english questionaire. (PDF) [file pone.0264901.s002.pdf]

Questionnaire and clinical data of patients hospitalized at the HUCAM-Covid 19 project.
Name:____ register:____________________
Phone number: ____
01. Date of birth: ___/___/_____ 02. Age:_______ 03. RESIDENCY:____________________
04. Ethinicitty: ( ) black ( ) other
05. Living with partner:_________________ 06. Schooling:____________________
07. Cohabitants:_________ 08. Elderly cohabitants:_______
09. Currently employed: _________________________________
10. Previous chronic disease: ( ) No ( ) Hypertension ( ) Other cardiovascular disease ( ) Diabetes ( ) lung disease ( ) kidney disease ( ) immunosuppressive disease ( ) Obesity ( ) STD ( ) other_______________________________________
Current weight: ___________ Hight: ___________ ( ) alcohol abuse ( ) other drug abuse: which one?___________ ( ) smoking: cigars/day___________ ( ) use of medicine: which?____________
11. Obstetric issues:
Pregnancies: ______ Births: _____ Abortions:______ Stillbirths: ________ Neonatal-death:______
Gestational Age: _________________ Blood Type: ___________________
Gestational Diabetes: ( ) yes ( ) no
Preeclampsia: ( ) yes ( ) no
Hyperemesis ( ) yes ( ) no
Rubella: IgG ( ) IgM ( )
Toxoplasmosis: IgG ( ) IgM ( )
HBsAg: ( ) positivo ( ) negativo
Anti HCV: ( ) positivo ( ) negativo
ANTI-HIV: ( ) positivo ( ) negativo
VDRL: ___________________
Other:____
13. Presented any of these symptoms since March 2020?
( ) Fever or chills
( ) Cough
( ) Shortness of breath
( ) Sore throat
( ) Headache
( ) Asthenia/myalgia
( ) Diarrhea
( ) Loss of sense of smell and taste
( ) Sneezing
(.) Runny nose
( ) None
14.1 If yes, When it began?_______________________________
14.2 When did the symptoms end?______________________________________
14. Have you been seen by a health professional in the last few months? (since March 2020)
( ) yes ( ) no
15. In the last months (since march 20) have you been hospitalized due to flu syndrome?
( ) yes, in the hospital ward ( ) yes, in the icu ( ) no
16. Have you been submitted to the rt-pcr test for sars-cov-2 detection?
( ) no ( ) yes, result:____________ when?_____________________________
17. Have you had close contact with a confirmed case of covid-19?? ( ) yes ( ) no
18. Did you have close contact with a suspected case of covid-19? ( ) yes ( ) no
19. Have you had any interstate or international travel in the past few months? ( ) yes ( ) no
21: Have you been working for the last few months? ( ) yes ( ) no ( ) remote
22: Did you comply with government guidelines to leave the house to perform only strictly necessary activities? ( ) yes ( ) no
23. Clinical examination on admission:
BP: ___/____mmHg. Edema: ( ) sim ( )não. Uterine fundus measurement: _____cm.
Metrosystoles: ( ) no ( ) yes ____/10min. Aminiorrexis: ( ) yes ( ) no.
Axillary temperature: _____ Respiratory frequency: _____ Heart rate _____ SpO2: ______
24. Exams performed on admission to hospital:
Covid-19 rapid test: IgG _______ IgM________
PCR para COVID-19 (until 48H): ( ) positive ( ) negative (.) not performed
HIV rapid test: ( ) positive ( ) negative
Syphilis rapid test: ( ) positive ( ) negative
Hepatitis B rapid test: ( ) positive ( ) negative

Hepatitis C rapid test: ( ) positive ( ) negative

25. Obstetric outcome: Date of birth:____/____/2020
25.1 ( ) term birth ( ) premature birth ( ) abortion ( ) molar pregnancy ( ) ectopic pregnancy
( ) other _________________________
25.2 ( ) spontaneous vaginal birth ( ) induced vaginal birth ( ) emergency cesarean birth
( ) elective cesarean birth.
25.3 Indication for delivery (induction/c-section):
____
25.4 Birth complications:____

26. NB data:
( ) AGA ( ) SGA ( ) LGA
Apgar index: _____/______/_______ ____
Birth weight: ____________________ **Length:** _____________ **TP**: ________ **CP:** _______ **AP:**_______
Cord clamping: ( ) opportune ( ) immediate
Rupture of membranes: ( ) at the time of expulsion ( ) before expulsion/c-section
Time since ruptured membranes: ____________
Neonatal ICU admission: ( ) yes ( ) no
Congenital malformation: ( ) yes ( ) no If yes, which one?_____________________________________
Birth trauma: ( ) yes ( ) no
Neonatal death: ( ) stillbirth ( ) postnatal death ( ) no
Hypoglycemia: ( ) yes ( ) no
Nb blood type: ______ ( )
Jaundice: ( ) yes ( ) no
Phototherapy: ( ) yes ( ) no
Weigh on hospital discharge: ______________g Days since birth:___________
NB condition at hospital discharge: ( ) exclusive breast-feeding ( ) BF + infant formula ( ) only formula

DATE:_____/______/2020.
