# Supplementary material for: SARS-CoV-2 infection in pregnant women assisted in a high-risk maternity hospital in Brazil: Clinical aspects and obstetric outcomes
Source: PLoS One. 2022 Mar 11;17(3):e0264901. doi: 10.1371/journal.pone.0264901 (PMC8916667; doi:10.1371/journal.pone.0264901)
Supplement: S1 Table — Specifies the gestational age at which each patient was pcr positive for SARS-CoV-2, the IgG and IgM result at hospital admission for the outcome (delivery or miscarriage), and the gestational age at the outcome. (PDF) [file pone.0264901.s003.pdf]

Gestational Age at PCR-positive test and presence of SARS-CoV-2 antibodies.

Patient	GA at PCR + (wk)	GA at delivery/miscarriage (wk)	IgM (delivery/outcome)	IgG (delivery/outcome)
**1**	30	38	Positive	Positive
**2**	33	36	Negative	Negative
**3**	37	37	Negative	Negative
**4**	26	32	Positive	Positive
**5**	32	37	Positive	Positive
**6**	20	37	Positive	Positive
**7**	17	33	Negative	Negative
**8**	19	38	Negative	Negative
**9**	25	39	Negative	Positive
**10**	26	38	Negative	Positive
**11**	17	41	Positive	Positive
**12**	39	39	Negative	Positive
**13**	38	38	Negative	Negative
**14**	26	40	Positive	Positive
**15**	29	37	Positive	Positive
**16**	8	15	Negative	Positive
**17**	28	37	Negative	Positive
**18**	33	37	Positive	Positive
**19**	31	39	Negative	Negative
**20**	34	38	Negative	Positive
**21**	32	39	Negative	Negative
**22**	23	34	Positive	Positive
**23**	26	38	Positive	Positive
**24**	38	38	Positive	Positive

**25**	25	39	Positive	Positive
**26**	31	39	Positive	Positive
**27**	25	37	Positive	Positive
**28**	38	38	Negative	Negative
**29**	28	38	Positive	Positive
**30**	27	41	Negative	Negative
**31**	26	38	Negative	Negative
**32**	8	8	Negative	Positive
**33**	23	23	Negative	Negative
**34**	37	37	Negative	Negative
**35**	33	33	Not performed	Not performed
**36**	30	30	Not performed	Not performed
**37**	29	29	Not performed	Not performed
**38**	37	37	Not performed	Not performed

GA: Gestational Age; PCR: Viral detection by reverse transcription polymerase chain reaction; wk: week.
